# Annotations

**Published:** 1909-02-06

**Authors:** 


### ANNOTATIONS.

### A Short Way with Quackery.

We learn from one of our lay contemporaries, which has especially distinguished itself for many years by unremitting onslaughts on all forms of quackery, some interesting particulars about the Quackery Prevention Act, which came into force on the first day of this year in New Zealand. It appears that a fine not exceeding £100 for a first offence, and not exceeding £200 for each subsequent offence, may be inflicted upon anyone who publishes or causes to be published any statement intended to promote the sale of medicines or other curative appliances which is false in any material particular. The sweeping nature of this most salutary provision will be realised when it is added that the printer, publisher, and proprietor are all liable, equally with the advertiser, for breaches of this law; a principle so obviously sound, and, indeed, vital, that it may be hoped the home legislature may some day take a leaf from the New Zealand statute book in this respect. The objection that the publisher and owner of a newspaper have no means of deciding whether any given statement about a medicine is true or false is met by a clause requiring that warning shall be delivered to them by the chief health officer on this point, and that they shall be liable only for the continued insertion of the false statements notified. Probably this of itself will effect much; a similar measure has long been urgently wanted in these islands. Journals of the highest repute, and deservedly so, as far as their literary contents are concerned, still accept in profusion the most shameless legends of the quackmonger. It requires only an intelligent public opinion and an anti-quackery law such as that of New Zealand, to convince the owners that their interests are best served by ceasing to connive at the spoliation and deception of their clientèle.

### The Doctor in Stageland.

Doctors of one kind or another, but more often than not of a very conventional kind, are quite common objects of stageland. The stage doctor is perhaps hardly as definite or as familiar a type as the stage lawyer; but in many plays he can be recognised by his manner, dress, and appearance, without reference to his title on the programme or to the words assigned to him. To the critical eye he many sometimes seem a trifle unsubstantial, and as a practitioner of medicine he may sometimes appear to lack the true professional touch. Medicine and the stage, indeed, have very pleasant associations, personal and professional. It is well known, of course, that Sir Charles Wyndham qualified as a medical practitioner before adorning the stage; and at the present moment Mr. Somerset Maugham, a comparatively junior member of our profession, is perhaps the most successful playwright of the day. In the course of his brilliant and meteoric career as a popular dramatist, Mr. Maugham did not at first draw directly for inspiration, either in plot or character, upon his medical experiences. In fact there was very little of the doctor about any of the three plays which took the theatre-going public by storm last year. But in "Penelope," his latest production, which is now drawing the town to the Comedy Theatre, Mr. Maugham has at length given direct evidence of first-hand knowledge of matters medical. Nevertheless even here he or someone else has obviously taken pains to adjust Dr. O'Farrell, the principal male character of the piece—at least in some degree —to the conventional pattern of the stage doctor. True, Mr. Maugham has allowed his doctor to abuse the privileges of his profession in order to carry on an intrigue with a lady who is ostensibly a patient, but in other respects the medical element in "Penelope" affords nothing but amusement, and is indeed the means of introducing characters and incidents conceived and executed in the author's drollest and most telling manner. Thus the doctor's widow, who only discloses her claim to gratuitous attention after O'Farrell has squandered much time and care upon her imaginary ailments, is vastly diverting, and keeps the house convulsed with laughter; while scarcely less realistic is the dreadful little hypochondriac who hears with regret that there is nothing the matter with him. "Penelope" is a very amusing entertainment for anyone, but especially for doctors.

### Climate.

The importance of climate in the treatment of certain forms of disease and of recovery from disease is no novelty to the medical profession or to the general public. Climatology as it affects the sick has been studied carefully by many physicians, and there are also text-books about it. But of climatology in its broader sense, the effects of local meteorological conditions upon the healthy, upon crops, domestic animals, industries, of the economic aspects of climatology in short, far too little account has hitherto been taken by political economists and statesmen. It is possible that the advances of tropical medicine of recent years are responsible for the awakening from this state of indifference; indeed, object-lessons on very striking scales are to be seen on all sides, from the Panama Canal downwards. At all events it is somewhat gratifying to notice that serious consideration is now not uncommonly devoted to questions of climate and its economic effects in the more important reviews. Just now, too, the "Clerk of the Weather," Dr. W. N. Shaw, is delivering at the London School of Economics, in Clare Market, a series of ten lectures on the climates of the possessions which constitute the British Empire. As a matter of fact, the serious study of meteorology dates back a comparatively short time even in the most civilised countries, and in many of our least developed colonies has not begun at all as yet. Very few places indeed have methodical records dating back half a century, and such statements on the climates of very many localities as have hitherto been published are only too often based upon general impressions rather than exact statistics. The serious study of climatology is undoubtedly still in its infancy, and our successors will enjoy far greater facilities than we do for the proper appreciation of this most important science and its teachings.

